# The Effect of an mHealth Self-Monitoring Intervention (MI-BP) on Blood Pressure Among Black Individuals With Uncontrolled Hypertension: Randomized Controlled Trial

**DOI:** 10.2196/57863

**Published:** 2024-06-28

**Authors:** Lorraine R Buis, Junhan Kim, Ananda Sen, Dongru Chen, Katee Dawood, Reema Kadri, Rachelle Muladore, Melissa Plegue, Caroline R Richardson, Zora Djuric, Candace McNaughton, David Hutton, Lionel P Robert, Sun Young Park, Phillip Levy

**Affiliations:** 1 Department of Family Medicine University of Michigan Ann Arbor, MI United States; 2 School of Information University of Michigan Ann Arbor, MI United States; 3 Department of Biostatistics University of Michigan Ann Arbor, MI United States; 4 Department of Emergency Medicine and Integrative Biosciences Center Wayne State University Detroit, MI United States; 5 Department of Pediatrics University of Michigan Ann Arbor, MI United States; 6 Department of Family Medicine Brown University Providence, RI United States; 7 Department of Medicine Sunnybrook Research Institute University of Toronto Toronto, ON Canada; 8 School of Public Health University of Michigan Ann Arbor, MI United States

**Keywords:** blood pressure, hypertension, mobile health, mHealth, mobile phone, smartphone

## Abstract

**Background:**

Hypertension is one of the most important cardiovascular disease risk factors and affects >100 million American adults. Hypertension-related health inequities are abundant in Black communities as Black individuals are more likely to use the emergency department (ED) for chronic disease–related ambulatory care, which is strongly linked to lower blood pressure (BP) control, diminished awareness of hypertension, and adverse cardiovascular events. To reduce hypertension-related health disparities, we developed MI-BP, a culturally tailored multibehavior mobile health intervention that targeted behaviors of BP self-monitoring, physical activity, sodium intake, and medication adherence in Black individuals with uncontrolled hypertension recruited from ED and community-based settings.

**Objective:**

We sought to determine the effect of MI-BP on BP as well as secondary outcomes of physical activity, sodium intake, medication adherence, and BP control compared to enhanced usual care control at 1-year follow-up.

**Methods:**

We conducted a 1-year, 2-group randomized controlled trial of the MI-BP intervention compared to an enhanced usual care control group where participants aged 25 to 70 years received a BP cuff and hypertension-related educational materials. Participants were recruited from EDs and other community-based settings in Detroit, Michigan, where they were screened for initial eligibility and enrolled. Baseline data collection and randomization occurred approximately 2 and 4 weeks after enrollment to ensure that participants had uncontrolled hypertension and were willing to take part. Data collection visits occurred at 13, 26, 39, and 52 weeks. Outcomes of interest included BP (primary outcome) and physical activity, sodium intake, medication adherence, and BP control (secondary outcomes).

**Results:**

We obtained consent from and enrolled 869 participants in this study yet ultimately randomized 162 (18.6%) participants. At 1 year, compared to the baseline, both groups showed significant decreases in systolic BP (MI-BP group: 22.5 mm Hg decrease in average systolic BP and *P*<.001; control group: 24.1 mm Hg decrease and *P*<.001) adjusted for age and sex, with no significant differences between the groups (time-by-arm interaction: *P*=.99). Similar patterns where improvements were noted in both groups yet no differences were found between the groups were observed for diastolic BP, physical activity, sodium intake, medication adherence, and BP control. Large dropout rates were observed in both groups (approximately 60%).

**Conclusions:**

Overall, participants randomized to both the enhanced usual care control and MI-BP conditions experienced significant improvements in BP and other outcomes; however, differences between groups were not detected, speaking to the general benefit of proactive outreach and engagement focused on cardiometabolic risk reduction in urban-dwelling, low-socioeconomic-status Black populations. High dropout rates were found and are likely to be expected when working with similar populations. Future work is needed to better understand engagement with mobile health interventions, particularly in this population.

**Trial Registration:**

ClinicalTrials.gov NCT02955537; https://clinicaltrials.gov/study/NCT02955537

**International Registered Report Identifier (IRRID):**

RR2-10.2196/12601

## Introduction

### Background

Hypertension affects >100 million American adults, which is nearly half of individuals aged ≥20 years [1]. Hypertension is also one of the most important cardiovascular disease risk factors, and when uncontrolled, it can cause adverse health outcomes such as myocardial infarction, stroke, heart failure, and chronic kidney disease [2-6]. Despite the importance of maintaining adequate blood pressure (BP) control, the American Heart Association has reported that only approximately 21.6% of those with hypertension have their BP controlled within age-adjusted criteria. Furthermore, 38.8% are unaware of their condition [1]. Hypertension-related health inequities are abundant in Black communities. Compared to White individuals, Black individuals have a greater prevalence of hypertension, hypertension-associated disease severity, and younger age of onset, making uncontrolled hypertension a significant problem in this population [7]. Moreover, Black individuals are more likely to use the emergency department (ED) for chronic disease–related ambulatory care, which is strongly linked to lower BP control, diminished awareness of hypertension, and adverse cardiovascular events [8-10].

Although uncontrolled hypertension is linked to a host of adverse outcomes, BP can typically be well controlled through lifestyle and behavior changes. Recommendations for managing hypertension have been consistent for decades and center on positive health behaviors such as maintaining a healthy weight, reducing daily sodium intake, increasing physical activity, and adhering to prescribed antihypertensive therapies [11]; however, engaging in these behaviors is difficult for many individuals, and this is especially true in Black individuals, who are less likely than White individuals to report adherence to preventive behaviors [1]. With the increasing national conversation focused on health inequities and social determinants of health [12], population-specific interventions are needed to intervene in communities where the burden of hypertension is disproportionately high.

### Objectives

Mobile health (mHealth) for chronic disease self-management is increasing in use, and based on this, as well as on the high penetration of smartphone ownership among Black individuals (currently approximately 83%) [13], we sought to develop and test an mHealth intervention (MI-BP). The MI-BP intervention was developed with the intention of educating and supporting self-monitoring of multiple health behaviors to reduce BP among Black individuals with uncontrolled hypertension recruited from urban EDs and community-based settings. Our goal was to determine the effect of MI-BP on the primary outcome of BP and secondary outcomes of physical activity, sodium intake, medication adherence, and BP control compared to enhanced usual care in a 1-year randomized controlled trial (RCT). We hypothesized that (1) mean systolic BP (SBP) would be significantly lower in the MI-BP arm than in the control group after 1 year (hypothesis 1) and (2) measures of physical activity, sodium intake, medication adherence, and BP control would be significantly better in the MI-BP arm than in the control arm after 1 year (hypothesis 2).

## Methods

### Overview

This study was a 1-year, 2-group RCT of the MI-BP intervention compared to enhanced usual care. The study was overseen by our Data and Safety Monitoring Board (DSMB). Details on the full study protocol have been previously published [14], but a summary of those procedures follows.

### Clinical Setting and Recruitment

All recruitment occurred in Detroit, Michigan, and was primarily conducted at the Detroit Medical Center in the EDs of Detroit Receiving Hospital and Sinai-Grace Hospital. Potentially eligible participants were screened by trained volunteers or by study staff members. Once a potentially eligible participant was identified according to clinical criteria, a research staff member spoke with the treating physician to determine whether they were a good candidate for participation. If so, individuals were informed of the study, screened further, and then consented and enrolled if they were interested and met the eligibility criteria. Additional recruitment occurred at community events where BP screening was conducted, such as mobile health unit visits, health fairs, and other health-related community events. Procedures for these potential participants were the same except for not checking with treating physicians to determine whether our staff should proceed with screening and enrollment.

### Eligibility Screening and Consent

#### Inclusion Criteria

To be eligible to participate in this trial, individuals were required to be Black, between the ages of 25 and 70 years, previously diagnosed with hypertension, have a smartphone compatible with the MI-BP intervention, and have uncontrolled BP (SBP >135 mm Hg) at triage and on repeat measurement using a BpTRU BPM-200 device (Smiths Medical PM, Inc) or Omron HEM 907XL IntelliSense (Omron Healthcare, Inc) at least 1 hour after triage vitals were taken.

#### Exclusion Criteria

Individuals were excluded from this trial if they were pregnant; had serious existing medical conditions that may make BP control difficult or necessitate frequent hospitalization (ie, previous diagnosis of resistant hypertension, steroid-dependent asthma or emphysema, cirrhosis or hepatic failure, stage-C or stage-D chronic heart failure, stage-IV or stage-V chronic kidney disease, and terminal cancer or ongoing active chemotherapy or radiation therapy); had a history of other serious medical conditions (eg, stroke, dementia, myocardial infarction, or known coronary artery disease); or had a history of alcohol or drug abuse as determined using the Cut down, Annoyed, Guilty, and Eye-opener Adapted to Include Drugs questionnaire (excluded if score was ≥2).

### Study Procedures

#### Baseline Data Collection Visit

After consent and enrollment, participants were scheduled for a return visit 1 to 2 weeks later for baseline data collection at a nearby university building. Transportation to all study visits via taxi or ride-sharing service was offered to anyone requiring transportation assistance. At the baseline visit, a secondary BP screening was conducted to ensure that we were only retaining participants with persistent uncontrolled hypertension in the study. At this time, participants who had an SBP of <130 mm Hg were deemed ineligible and excluded from the study. Next, baseline data were collected. To control response fatigue in the baseline data collection survey, we created 6 different permutations, each with a different order of instruments, which were also balanced within blocks. At this time, participants were also given a prescription for antihypertensive therapy. If needed, referrals to primary care were made by study physicians. In the event that a participant was already taking antihypertensive medications prescribed through a preexisting relationship with a primary care provider (PCP), we contacted their PCP to inform them of our algorithm-based approach to antihypertensive therapy and coordinated with them when medication adjustments were indicated.

#### Medication Titration and Randomization Visit

At 2 weeks after the baseline visit, participants were assessed for medication titration, the process of adjusting antihypertensive medication dosages to ensure appropriate and optimal treatment. At this time, participants were randomized into 1 of the 2 study arms in the trial. In total, it took approximately 4 weeks for an enrolled participant to be randomized into the study. This month-long de facto washout period was designed to ensure that we were truly reaching individuals with uncontrolled hypertension and who were not just temporarily presenting with elevated BP in the ED. Moreover, our previous experiences conducting work in this setting demonstrated high levels of attrition between ED recruitment and initial follow-up. Delaying randomization also helped ensure identification of individuals who did not intend to fully participate at the outset, increasing the likelihood of randomized participant retention. Trial randomization was stratified by sex in blocks of equal size. Study staff responsible for arm allocation were blinded to block size to prevent contamination. After randomization, all study materials, including any equipment, were distributed to the participants according to the treatment arm. A second titration visit was conducted 6 weeks after randomization, and the need for titration was assessed at each subsequent follow-up visit.

#### Quarterly Follow-Up Visits

Data collection assessments were conducted at weeks 0, 13, 26, 39, and 52 using a consistent set of study measures. In addition to survey measures, patients were instructed to bring their hypertension medications with them so pill counts could be conducted. As electronic health record data were not available to our study team, all medication data, including prescribed medication names and doses, were self-reported or captured from pill bottles. We also monitored for any potentially harmful renal or metabolic issues at baseline and weeks 26 and 52 and adjusted medications accordingly. To measure sodium intake (a secondary outcome measure of interest), at weeks 0, 26, and 52, participants were given supplies to collect 24-hour urine for sodium measurement. Study staff collected these specimens directly from the participants at their home to improve adherence. All medication titration and study follow-up visits were free; however, participants were responsible for the cost of medications, PCP visits, or copays, as applicable.

#### Impact of COVID-19 on Study Procedures

In March 2020, the MI-BP trial was closed to new enrollments and in-person data collection due to the COVID-19 pandemic. This necessitated protocol changes in the following weeks and months in an effort to maximize data collection from participants who were enrolled in the study before the pandemic. To summarize these changes, we pivoted to remote data collection for follow-up assessments via phone or videoconference. This meant that home-monitored BP measurements using study-issued cuffs served as the final outcome measures for participants completing their trial participation between March 2020 and April 2021. In addition, as all in-person participant interaction had been suspended, all laboratory measures were discontinued during the COVID-19 pandemic, and survey-based assessments were conducted verbally by phone or videoconference out of concern for literacy levels among participants. We also removed several instruments from interim follow-up assessments in weeks 13 and 39. Finally, anthropometric assessments, including weight, height, and waist circumference measurements, were self-reported by participants using their own home scales and tape measures. Given the increased reliance on home-based, self-reported data, the chance of missing data from follow-up assessments was greater.

### Trial Arms

Participants in this trial were randomized equally to 1 of the 2 treatment arms, which included an enhanced usual care control arm and the MI-BP intervention arm.

#### Enhanced Usual Care (Control Arm)

Participants in the enhanced usual care group were given a prescription for antihypertensive medications, printed educational materials on hypertension, and a BP monitor for daily use. Participants assigned to the enhanced usual care control group received no further intervention; however, they were asked to take part in all study-specific follow-up visits. The decision to provide home BP cuffs to control participants, above and beyond true usual care, was made to reflect the fact that home BP monitoring is widely accepted as a guideline-based standard of care for individuals being treated for hypertension [6], making it appropriate to include in the usual care arm. We acknowledge that this active control represents a departure from true usual care; however, it does represent an ideal usual care scenario based on current hypertension management guidelines.

#### MI-BP (Treatment Arm)

Participants randomized to receive the MI-BP intervention were given a prescription for antihypertensive medication, a Bluetooth-enabled pedometer (Fitbit Zip), a BP cuff, and access to the MI-BP mobile app. Participants were asked to use the MI-BP mobile app and related peripheral devices for 12 months.

#### MI-BP Intervention

##### Overview

MI-BP is a comprehensive, multicomponent intervention that targets multiple behaviors for managing hypertension via smartphone app, including BP self-monitoring, physical activity tracking, sodium intake tracking, goal setting, educational and motivational messaging, and medication adherence reminders. The MI-BP app was developed by Vibrent Health, a digital health company. Vibrent Health designed the app, study staff web-based portal, and server platforms necessary to support this trial. The MI-BP app was previously described in detail but is summarized in this section [14].

##### BP Monitoring

To support BP self-monitoring, participants who could use a standard BP cuff (suitable for an arm circumference between 23 and 45 cm) were provided with a Bluetooth-enabled BP cuff (A&D UA-651BLE) that could sync to the MI-BP app. The MI-BP app showed different visualizations of BP over time, including both graph and log form. In the event that a participant required a larger cuff size (between 42 and 60 cm), we provided an extra-large arm monitor (A&D LifeSource UA-789), which was not Bluetooth enabled and required manual data entry. Participants were instructed to measure and sync (or manually enter) their BP to the MI-BP app at home using a commonly accepted home BP-monitoring protocol for a minimum of 3 days per week; however, daily self-monitoring and syncing were encouraged. If participants self-monitored an SBP reading of >180 or <100 mm Hg or a diastolic BP (DBP) reading of >110 mm Hg, they were instructed by the study staff at baseline, as well as by automated notifications within the app at the time of the elevated reading, to check their BP again. If it was still elevated after 3 days, participants were instructed to call the study staff. Participants were also instructed to report to the ED and follow up afterward with a call to the research staff if they were experiencing symptoms of dizziness, chest pain, severe headache, vision changes, or numbness or weakness in the face or extremities.

##### Physical Activity Monitoring and Tracking

To support physical activity self-monitoring, participants were provided with a Fitbit Zip pedometer that could sync to the MI-BP app, which showed different visualizations (graph and log form) of physical activity data over time. Participants were instructed to wear their Fitbit daily and sync the device at least once per week.

##### Sodium Intake Monitoring and Tracking

To support sodium intake monitoring, the MI-BP app used a logging approach that encouraged participants to identify their intake of high-sodium foods using a checklist-type log available within the MI-BP app. The checklist comprised 7 categories with 3 to 8 items per category and represented the most common types of high-sodium foods that contribute to high-sodium diets. Although we encouraged users to track their intake of high-sodium foods daily, users were instructed to engage at a minimum in highly focused, 3-day consecutive bouts of logging that were prompted within the MI-BP app.

##### Goal Setting

Participants received weekly step count goals that were displayed in the MI-BP app and were also delivered via push notifications. On the basis of previous work from our team [15-19], step count goals were gradually incremented and were based on an average of 7 consecutive days of data, during which at least 5 of the days needed to be considered valid. A valid day was defined as >200 steps per day. As we gradually incremented weekly goals, calculated goals never exceeded 600 additional steps over the previous goal. This gradual increment in weekly step count goals was made in an effort to reduce potential adverse events (AEs).

Goal setting for sodium intake was also conducted every 2 to 4 weeks after an intensive 1-week baseline self-monitoring period that was used to calculate the initial goal for each participant. Sodium intake goals were displayed within the app and were also sent via push notifications. Participants were instructed to log their intake of high-sodium foods for a 3-day period approximately 2 weeks after receiving their initial sodium intake goal that limited the number of high-sodium foods to be consumed. When sodium goals were met during a logging period, a new lower sodium intake goal was issued, and participants were asked to log their intake of high-sodium foods 4 weeks later. If the goal was not met, participants were asked to try again in 2 weeks. Additional details on our sodium logging and goal-setting protocols have been published previously [14].

##### Messaging

MI-BP provided users with 4 different types of messages, which were sent via push notifications and in-app messaging. These included educational messaging focused on hypertension, physical activity, sodium intake, and tips for behavior change and overcoming barriers to behavior change; motivational messaging; tailored messaging, including tips for overcoming specific self-reported barriers to behavior change and daily medication reminders as well as tailored feedback responsive to whether participants were meeting their set goals; and customizable daily medication reminders. In addition to the customizable daily medication reminders, MI-BP sent approximately 7 messages per week. Message content, frequency, and timing were varied and tailored wherever possible to maximize user engagement.

#### Measures

We collected a variety of measures throughout this study. Full details of our study measures have been published previously [14]; however, those discussed in this paper are described in this section. Data were collected at baseline; medication titration visits at weeks 2 and 8; and planned follow-up assessments at weeks 13, 26, 39, and 52. Although most measures were collected at all time points, some were collected less frequently due to participant burden and cost of administration. The primary outcome measure of BP was collected at the clinic (or at home with study issued BP cuffs during the COVID-19 pandemic) and was assessed at every study visit by a trained study staff member using a BpTRU BPM-200 or Omron HEM 907XL IntelliSense BP-monitoring device. Secondary outcome measures included the following: physical activity as measured using the International Physical Activity Questionnaire–Short Form (IPAQ-SF) [20]; sodium intake as measured using the Block Sodium Screener (BSS) [21] as well as a 24-hour urine sodium test; self-reported medication adherence using the Adherence to Refills and Medication Scale (ARMS-14) [22]; and self-efficacy for changing targeted behaviors, including physical activity via the Self-Efficacy for Exercise Behaviors (SEEB) scale [23] and medication adherence via the Medication Adherence Self-Efficacy Scale (MASES) [24], as well as diet using an investigator-developed 11-item instrument assessing confidence in reducing sodium consumption, avoiding high-fat foods, avoiding sugar-sweetened beverages, and improving vegetable and legume intake. Additional measures included hypertension knowledge measured by the Hypertension Evaluation of Lifestyle and Management (HELM) scale [25]; health literacy measured by the Rapid Assessment of Adult Literacy in Medicine–Short Form (REALM-SF) [26]; patient activation measured by the Patient Activation Measure (PAM) [27], and health-related quality of life measured by the Short Form–12 (SF-12) [28]. In addition to instruments assessing physical activity, sodium intake, and clinic-measured BP, we analyzed related study data from MI-BP treatment arm participants collected in the app.

### Statistical Analysis

#### Sample Size

As stated in our study protocol [14], our sample size was initially developed with 2 co–primary outcomes: SBP measured continuously and SBP control (defined dichotomously as either above or below the SBP target of 130 mm Hg), which is a more conservative measure. After experiencing sustained challenges with recruitment, the more conservative dichotomous BP control measure was dropped as a co–primary outcome. This necessitated a recalculation in sample size based solely on continuously measured SBP [29]. Due to a lack of similar studies available at the time, we estimated a drop of 10 and 17 mm Hg points in SBP in the usual care and MI-BP arms, respectively, at the end of the trial, based on estimates derived from our own previous work [30]. A constant between-subject SD of 10 mm Hg was assumed, along with an intrasubject correlation of 0.5 [31]. With 121 participants per arm, these estimates would allow us to detect a group-by-time interaction with power of >95% at a 5% level of significance. Allowing for 20% attrition, we sought to recruit 152 participants per study arm for a total of 304 participants.

#### Analysis Plan

Descriptive statistics were calculated for demographic variables and baseline measures. To ensure balance across study arms, these measures were compared using 2-tailed *t* tests, Wilcoxon rank sum tests, or chi-square tests, as appropriate. Linear mixed models were used to investigate differences in change in the outcomes between study arms, with time, study arm, and their interaction as primary covariates. For all outcomes, time was entered as a categorical covariate. The models were further adjusted for age and sex. For the BP outcomes (SBP and DBP), a second set of models was explored, where time was entered as a linear term to capture the rate of change in the outcomes. All models included a random intercept to account for intrasubject correlation. Square root transformations were used for the IPAQ-SF and BSS, and a log transformation was applied to the ARMS-14 before running the linear mixed models to better meet model assumptions.

To investigate whether dropout was associated with any covariates, a time-to-dropout analysis was carried out using a Cox regression model. As this study was partially conducted during the COVID-19 pandemic, we wanted to consider the impact of COVID-19 on dropout. To that end, we defined a new variable, *COVID group*, for each individual at each time point. If the event time (eg, week 13) of individual assessments was before 3 PM on March 16, 2020, we considered these records as *Before COVID-19*. Otherwise, we considered the records as *During COVID-19*. Thus, the COVID-19 group was modeled as a time-dependent covariate in the Cox model. All statistical analyses were carried out in SAS (version 9.4; SAS Institute).

### Ethical Considerations

The methods for this study were approved by the institutional review boards (IRBs) at both Wayne State University (WSU; IRB 040416M1F) and the University of Michigan (HUM00114202). All participants provided written informed consent before enrollment. Participants received financial incentives to take part in this study, with each visit individually incentivized, and could earn up to US $275 over the course of 1 year. To protect the privacy and confidentiality of participants, all data are reported in the aggregate and no identifiable information is presented here.

## Results

### Trial Recruitment

**Figure 1:**
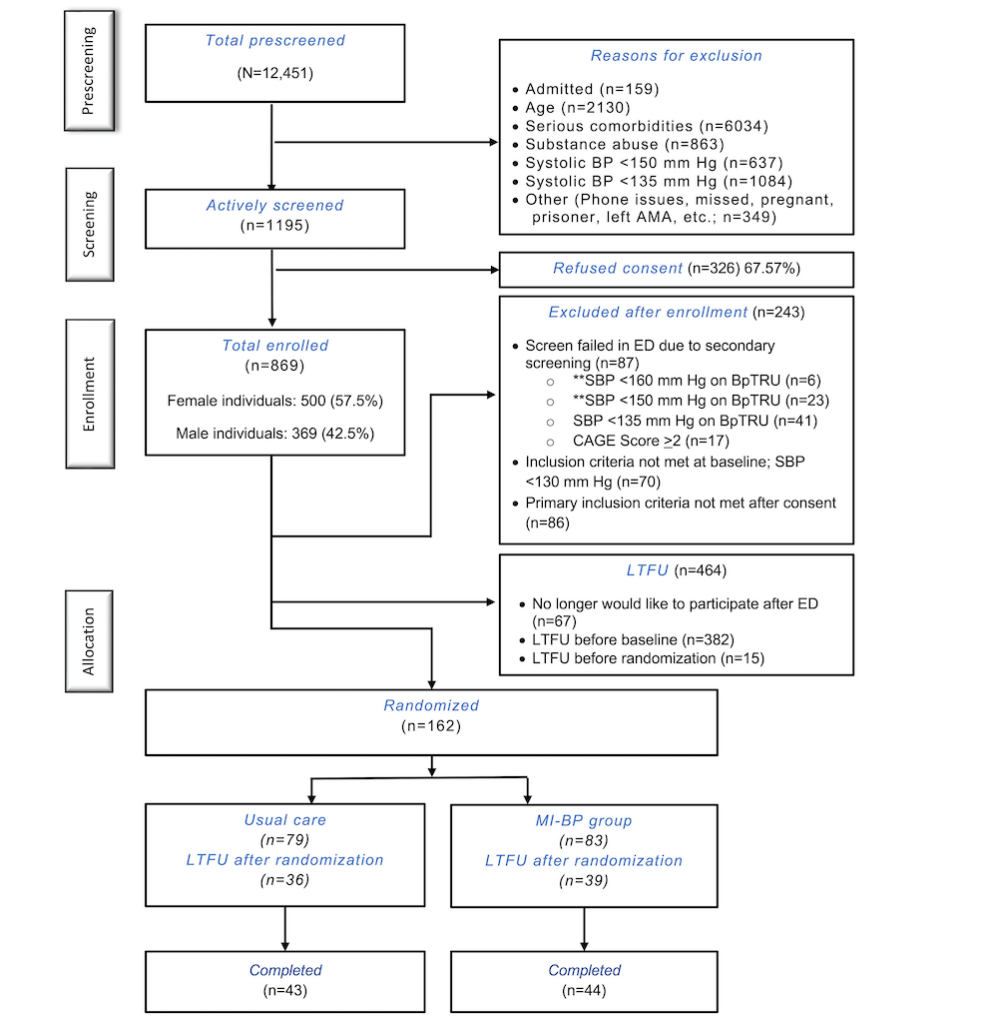
CONSORT (Consolidated Standards of Reporting Trials) diagram showing participant recruitment and retention. **Reflects secondary blood pressure (BP) criteria in the previous protocol. AMA: against medical advice; CAGE: Cut down, Annoyed, Guilty, and Eye-opener; ED: emergency department; LTFU: lost to follow-up; mHealth: mobile health; SBP: systolic blood pressure.

In total, we prescreened 12,451 individuals, predominantly in ED settings (n=12,089, 97.09% in EDs; n=169, 1.36% from community events; and n=193, 1.55% from mobile health units), of whom 1195 (9.6%) were preliminarily eligible for participation. Of those 1195 participants, 869 (72.72%) were consented and enrolled in this study. Most enrolled participants were excluded after enrollment (before randomization) for failing the secondary screening in the ED, not meeting the inclusion criteria at baseline, or not meeting the primary inclusion criteria after consent (243/869, 28%); lost to follow-up before randomization (416/869, 47.9%); or scheduled for baseline or randomization visits that were halted due to the COVID-19 pandemic (48/869, 5.5%). Figure 1 shows the CONSORT (Consolidated Standards of Reporting Trials) diagram of participant flow through the trial. Due to the COVID-19 pandemic, in March 2020, ED recruitment for the trial was suspended precluding new enrollments from the ED. While we were able to eventually transition to community-based recruitment using mobile health units under an IRB-approved protocol amendment within 9 months of this, screening was severely reduced, and no new randomizations occurred. These considerations, combined with the challenge of keeping participants engaged using remote follow-up, prompted a decision by the study team, made in conjunction with our DSMB, to end recruitment for this study in January 2022. Ultimately, we randomized 162 participants to the MI-BP trial.

### Participant Characteristics

The 162 participants randomized to this trial (n=79, 48.8% to usual care and n=83, 51.2% to the MI-BP intervention) were predominantly female (n=97, 59.9%) and were, on average, aged 48.3 (SD 9.3; range 29-68) years. As race was an inclusion criterion, 100% (162/162) of our participants were Black individuals. Participants were characterized by being single (86/162, 53.1%) and employed (97/162, 59.9%) and having a high school education or lower (90/162, 55.6%) and an average household income of <US $25,000 (73/162, 45.1%). Table 1 shows participant characteristics as well as summary baseline measures stratified by study arm.

**Table 1:** Participant characteristics and baseline measures by study arm (N=162).

Participant characteristics and baseline measures		Overall	Intervention (n=83)	Control (n=79)	*P* value^a^	
**Site, n (%)**						.45
	COM^b^	17 (10.5)	10 (12)	7 (8.9)		
	DRH^c^	83 (51.2)	45 (54.2)	38 (48.1)		
	SGH^d^	62 (38.3)	28 (33.7)	34 (43)		
**Sex, n (%)**						.82
	Female	97 (59.9)	49 (59)	48 (60.8)		
	Male	65 (40.1)	34 (41)	31 (39.2)		
Age (y), mean (SD)		48.33 (9.28)	48.47 (8.97)	48.18 (9.64)	.84	
Height (cm), mean (SD)		170.92 (10.16)	170.82 (10.85)	171.03 (9.46)	.95	
Weight (kg), mean (SD)		101.80 (26.48)	100.95 (26.49)	102.67 (26.60)	.82	
Waist circumference (mm), mean (SD)		1125.66 (203.53)	1102.4 (215.93)	1150.69 (188.83)	.29	
Heart rate (beats/min), mean (SD)		78.09 (13.73)	78.59 (13.23)	77.56 (14.30)	.63	
**Marital status, n (%)**						.81
	Single or never been married	86 (53.1)	42 (50.6)	44 (55.7)		
	Married or cohabitating	33 (20.4)	18 (21.7)	15 (19)		
	Divorced, widowed, or separated	43 (26.5)	23 (27.7)	20 (25.3)		
**Education, n (%)**						.86
	Lower than college	90 (55.6)	47 (56.6)	43 (54.4)		
	Some college	40 (24.7)	19 (22.9)	21 (26.6)		
	A college or technical degree	32 (19.8)	17 (20.5)	15 (19)		
**Insurance, n (%)**						.81
	Private health insurance	48 (29.6)	24 (28.9)	24 (30.4)		
	Medicare	19 (11.7)	10 (11.4)	9 (12.1)		
	Medicaid	67 (45.7)	37 (44.6)	30 (38.0)		
	No insurance	27 (16.7)	12 (14.5)	15 (19)		
	Unknown or refused to answer	1 (0.6)	0 (0)	1 (1.3)		
**Employment status, n (%)**						.33
	Currently employed	97 (59.9)	53 (63.9)	44 (55.7)		
	Others	64 (39.5)	30 (36.1)	34 (43)		
	Unknown or refused to answer	1 (0.6)	0 (0)	1 (1.3)		
**Annual household income (before taxes; US $), n (%)**						.42
	<10,000	45 (27.8)	24 (28.9)	21 (26.6)		
	10,000-24,999	28 (17.3)	16 (19.3)	12 (15.2)		
	25,000-49,999	36 (22.2)	21 (25.3)	15 (19)		
	≥50,000	14 (8.6)	11 (13.3)	3 (3.8)		
	Unknown, refused to answer, or missing	39 (24.1)	11 (13.3)	28 (35.4)		
**Health literacy (measured using the REALM-SF^e^), n (%)**						.43
	0 (third grade and below)	4 (2.5)	1 (1.2)	3 (3.8)		
	1-3 (fourth-sixth grade)	13 (8)	5 (6)	8 (10.1)		
	4-6 (seventh-eighth grade)	49 (30.2)	24 (28.9)	25 (31.7)		
	7 (high school)	95 (58.6)	53 (63.9)	42 (53.2)		
	Missing	1 (0.6)	0 (0)	1 (1.3)		
Hypertension knowledge (measured using the HELM^f^), mean (SD)		8.38 (2.37)	8.62 (2.27)	8.11 (2.47)	.19	
Patient activation (measured using the PAM^g^), mean (SD)		64.00 (12.67)	64.50 (13.50)	63.48 (11.81)	.60	
Health-related quality of life–Physical (measured by SF-12^h^ PCS^i^), mean (SD)		42.05 (11.88)	42.23 (11.26)	41.86 (12.57)	.98	
Health-related quality of life–Mental (measured by SF-12 MCS^j^), mean (SD)		48.85 (11.75)	50.18 (11.07)	47.46 (12.34)	.18	

^a^Descriptive statistics were calculated for demographic variables and baseline measures and compared across the study arms using 2-tailed *t* tests, Wilcoxon rank sum tests, or chi-square tests, as appropriate.

^b^COM: community-based recruitment.

^c^DRH: Detroit Receiving Hospital.

^d^SGH: Sinai-Grace Hospital.

^e^REALM-SF: The Rapid Estimate of Adult Literacy in Medicine–Short Form.

^f^HELM: Hypertension Evaluation of Lifestyle and Management Knowledge Scale.

^g^PAM: Patient Activation Measure.

^h^SF-12: Short Form–12 Health Survey.

^i^PCS: Physical Component Summary.

^j^MCS: Mental Component Summary.

### Effect of MI-BP on Outcomes

#### BP Outcome

**Figure 2:**
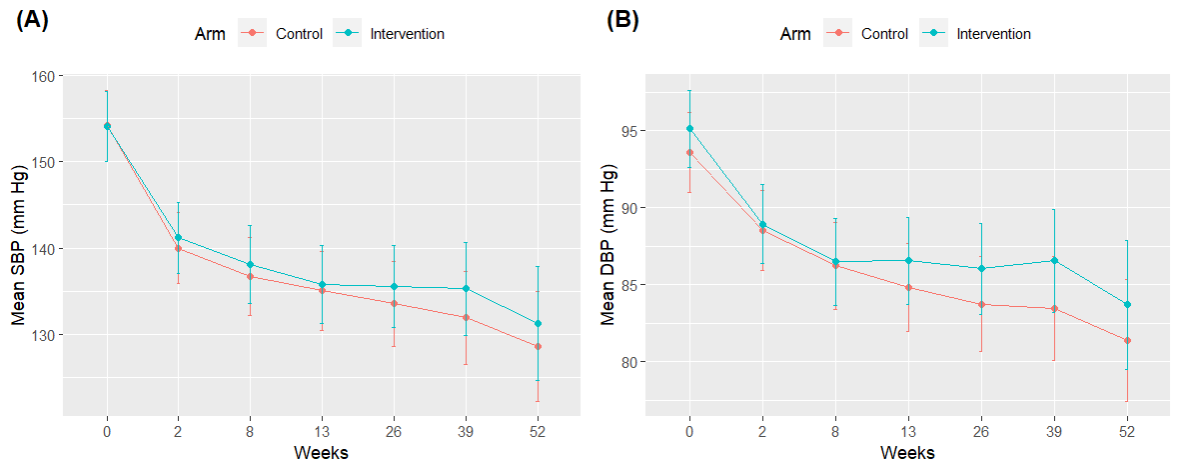
Unadjusted mean trajectories (with 95% confidence interval) for (A) systolic blood pressure (SBP) and (B) diastolic blood pressure (DBP) by study arm.

For our primary outcome of SBP, sex- and age-adjusted average baseline SBP was comparable between the groups (MI-BP group mean 153.92 mm Hg, SD 2.10; enhanced usual care group mean 153.96 mm Hg, SD 2.15; *P*=.99). Both groups saw a mostly steady and similar decline in SBP over the 12-month intervention (unadjusted means shown in Figure 2A). Table 2 shows a model-based assessment of pairwise differences in adjusted mean SBP for each study arm. At week 52, compared to baseline, the MI-BP group exhibited a 22.5 mm Hg decrease (SE 3.35 mm Hg; *P*<.001), and the control group exhibited a 24.12 mm Hg decrease (SE 3.25 mm Hg; *P*<.001) in average estimated SBP adjusted for age and sex. The average declines were not significantly different between the groups (time-by-arm interaction: *P*=.99). A regression model with a linear time term estimated the rate of decline in the MI-BP group to be 0.3 mm Hg per week (SE 0.05 mm Hg/wk; *P*<.001) and 0.34 mm Hg per week (SE 0.05 mm Hg/wk; *P*<.001) in the control group adjusted for age and sex. Again, these rates were not significantly different between the groups (interaction: *P*=.60; Table S1 in Multimedia Appendix 1). At week 52, overall 58% (19/33) of participants in the intervention group had controlled BP (secondary outcome, defined as SBP <130 mm Hg), whereas 53% (18/34) of participants in the nonintervention group achieved BP control. This difference was not statistically significant (*P*=.89).

**Table 2:** Estimated pairwise mean differences and SEs across time for systolic blood pressure by study arm.

Comparison	Intervention		Control	
	Mean difference (mm Hg, time 2 minus time 1) (SE)	*P* value	Mean difference (mm Hg, time 2 minus time 1) (SE)	*P* value
0 vs 2 weeks	–12.8394 (2.4156)	<.001	–14.1392 (2.4466)	<.001
0 vs 8 weeks	–15.3359 (2.5677)	<.001	–16.7350 (2.5790)	<.001
0 vs 13 weeks	–17.9052 (2.5566)	<.001	–18.6283 (2.6044)	<.001
0 vs 26 weeks	–17.4752 (2.6346)	<.001	–19.2175 (2.7335)	<.001
0 vs 39 weeks	–17.3283 (2.8752)	<.001	–21.2452 (2.8972)	<.001
0 vs 52 weeks	–22.5007 (3.3471)	<.001	–24.1173 (3.2502)	<.001
2 vs 8 weeks	–2.4966 (2.5852)	.33	–2.5958 (2.5790)	.31
2 vs 13 weeks	–5.0658 (2.5742)	.05	–4.4891 (2.6044)	.09
2 vs 26 weeks	–4.6358 (2.6520)	.08	–5.0782 (2.7335)	.06
2 vs 39 weeks	–4.4890 (2.8882)	.12	–7.1060 (2.8972)	.01
2 vs 52 weeks	–9.6613 (3.3583)	.004	–9.9781 (3.2502)	.002
8 vs 13 weeks	–2.5692 (2.6855)	.34	–1.8934 (2.6886)	.48
8 vs 26 weeks	–2.1393 (2.7702)	.44	–2.4825 (2.8147)	.38
8 vs 39 weeks	–1.9924 (2.9898)	.51	–4.5102 (2.9664)	.13
8 vs 52 weeks	–7.1647 (3.4428)	.04	–7.3823 (3.3121)	.03
13 vs 26 weeks	0.4300 (2.7428)	.88	–0.5891 (2.8296)	.84
13 vs 39 weeks	0.5768 (2.9774)	.85	–2.6169 (2.9850)	.38
13 vs 52 weeks	–4.5955 (3.4361)	.18	–5.4890 (3.3253)	.10
26 vs 39 weeks	0.1469 (3.0223)	.96	–2.0278 (3.0707)	.51
26 vs 52 weeks	–5.0255 (3.4733)	.15	–4.8999 (3.3983)	.15
39 vs 52 weeks	–5.1723 (3.6255)	.15	–2.8721 (3.5034)	.41

DBP exhibited a very similar pattern to that of SBP (Figure 2B), with the MI-BP and control arms experiencing significant reductions in DBP from baseline to 52 weeks with a 10.20 mm Hg (SE 1.82 mm Hg; *P*<.001) and 11.44 mm Hg (SE 1.75 mm Hg; *P*<.001) estimated average decrease, respectively (Table 3). However, no significant differences were found between the groups (time-by-arm interaction: *P*=.79). Model-based rates of decline were observed in the MI-BP (estimate=0.13 mm Hg/wk; SE 0.03 mm Hg/wk; *P*<.001) and control (estimate=0.17 mm Hg/wk; SE 0.03 mm Hg/wk; *P*<.001; Table S1 in Multimedia Appendix 1) groups. Again, none of these changes were statistically significantly different between the groups (*P*=.21).

**Table 3:** Estimated pairwise mean differences and SEs across time for diastolic blood pressure by study arm.

Comparison	Intervention		Control	
	Mean difference (mm Hg, time 2–time 1) (SE)	*P* value	Mean difference (mm Hg, time 2–time 1) (SE)	*P* value
0 vs 2 weeks	–6.2249 (1.2940)	<.001	–5.0759 (1.3099)	.001
0 vs 8 weeks	–7.9787 (1.3772)	<.001	–6.8315 (1.3829)	<.001
0 vs 13 weeks	–8.0509 (1.3713)	<.001	–8.1532 (1.3967)	<.001
0 vs 26 weeks	–8.2251 (1.4136)	<.001	–8.8694 (1.4665)	<.001
0 vs 39 weeks	–7.5165 (1.5436)	<.001	–9.5521 (1.5551)	<.001
0 vs 52 weeks	–10.1987 (1.8209)	<.001	–11.4393 (1.7453)	<.001
2 vs 8 weeks	–1.7538 (1.3861)	.21	–1.7555 (1.3829)	.20
2 vs 13 weeks	–1.8261 (1.3803)	.19	–3.0772 (1.3967)	.03
2 vs 26 weeks	–2.0003 (1.4225)	.16	–3.7935 (1.4665)	.01
2 vs 39 weeks	–1.2916 (1.5500)	.41	–4.4762 (1.5551)	.004
2 vs 52 weeks	–3.9738 (1.8263)	.03	–6.3634 (1.7453)	<.001
8 vs 13 weeks	–0.0723 (1.4389)	.96	–1.3217 (1.4403)	.36
8 vs 26 weeks	–0.2465 (1.4854)	.87	–2.0380 (1.5087)	.18
8 vs 39 weeks	0.4622 (1.6035)	.77	–2.7206 (1.5904)	.09
8 vs 52 weeks	–2.2200 (1.8703)	.24	–4.6078 (1.7768)	.01
13 vs 26 weeks	–0.1742 (1.4698)	.91	–0.7163 (1.5164)	.64
13 vs 39 weeks	0.5345 (1.5967)	.74	–1.3989 (1.6003)	.38
13 vs 52 weeks	–2.1477 (1.8669)	.25	–3.2861 (1.7837)	.07
26 vs 39 weeks	0.7087 (1.6201)	.66	–0.6827 (1.6455)	.68
26 vs 52 weeks	–1.9735 (1.8858)	.30	–2.5699 (1.8219)	.16
39 vs 52 weeks	–2.6822 (1.9658)	.17	–1.8872 (1.8775)	.32

#### Physical Activity

Slight improvements in physical activity over the course of the trial were found for both the MI-BP and enhanced usual care groups as measured using the iPAQ-SF, although the improvements were not statistically significant in general. In the MI-BP group, the age- and sex-adjusted average IPAQ-SF score (after square root transformation) increased by 10.25 metabolic equivalent of task (MET) minutes per week (SE 6.37 MET min/wk; *P*=.11) at 52 weeks, whereas the increase was 10.57 MET minutes per week in the control group (SE 5.87 MET min/wk; *P*=.07; Table S2 in Multimedia Appendix 1). Both groups exhibited fluctuations in the change pattern, where the up-and-down behavior was more prominent in the MI-BP arm (Figure S1 in Multimedia Appendix 2). However, there were no significant differences in the change pattern across the groups (time-by-group interaction: *P*=.93).

#### Sodium Intake

Figure S2 in Multimedia Appendix 2 shows that mean sodium intake measured using the BSS declined fairly steadily in the MI-BP arm, whereas in the enhanced usual care group, there was a fluctuating pattern in the mean trajectories. However, both arms experienced significant improvements when comparing the baseline with the 52-week values. In the MI-BP group, the average decrease in the adjusted (square root–transformed) BSS score was 0.36 (SE 0.19; *P*=.06), whereas the average decrease in the control arm was 0.60 (SE 0.18; *P*=.001; Table S3 in Multimedia Appendix 1). No significant time-by-group interaction was observed (*P*=.19). We successfully obtained 24-hour urine sodium samples from 136 participants at baseline and 32 participants at 52-week follow-up. In contrast to BSS results, no differential improvements in sodium concentration in 24-hour urine sodium samples were found across arms (time-by-group interaction, *P*=.56). 

#### Medication Adherence

Both treatment groups experienced significant improvements in medication adherence as measured using the ARMS-14 over 1 year (Figure S3 in Multimedia Appendix 2). The average estimated decrease at 52 weeks compared to the baseline in the log-transformed ARMS-14 score in the MI-BP group was 0.20 (SE 0.04; *P*<.001), whereas the corresponding decrease in the control arm was 0.15 (SE 0.04; *P*<.001; Table S4 in Multimedia Appendix 1). No significant difference in the pattern of change was observed (time-by-group interaction: *P*=.30).

#### Self-Efficacy

Self-efficacies were measured in 4 different ways related to attitude and habits of exercise, medication adherence, and eating habits. Figures S4A and S4B in Multimedia Appendix 2 show that the trajectories of exercise self-efficacy as measured using 2 subscales of the Self-Efficacy for Exercise Behaviors scale (Sticking to It and Making Time for Exercise) were similar in both study arms. However, in neither arm did the average scores change significantly. In the MI-BP arm, the average estimated decrease in the Sticking to It subscale score at week 52 from the baseline was 0.11 (SE 0.20; *P*=.59), whereas the corresponding decrease in the control arm was 0.01 (SE 0.18; *P*=.94). For the Making Time for Exercise subscale score, the adjusted mean decrease was 0.09 (SE 0.22; *P*=.69) in the MI-BP arm and 0.11 (SE 0.20; *P*=.58) in the control arm (Tables S5 and S6 in Multimedia Appendix 1). As with other outcomes, no significant time-by-group interactions were found (*P*=.88 and *P*=.94 for Sticking to It and Making Time for Exercise, respectively).

For medication adherence self-efficacy (MASES), statistically significant improvements were observed in both arms as the trajectories seemed to follow similar patterns (Figure S4C Multimedia Appendix 2). The increase in the estimated average MASES score in the MI-BP arm was 0.38 (SE 0.16; *P*=.02), and the corresponding increase in the control arm was 0.37 (SE 0.15; *P*=.02; Table S7 in Multimedia Appendix 1). No significant time-by-arm interaction was observed, suggesting no differences in the pattern of change between the groups (overall time-by-group interaction: *P*=.47).

Self-efficacy for eating behaviors showed worse values compared to the baseline (estimated mean 43.95) in the intervention group at week 52 as the estimated mean decreased from a baseline value of 43.95 to 40.04 at week 52 (estimated mean reduction 3.91; SE 1.47; *P*=.008). In contrast, in the control arm, the score increased slightly from baseline (mean 41.64) to week 52 (mean 43.69), although this improvement was not statistically significant (estimated mean improvement 2.05; SE 1.4; *P*=.14; Table S8 in Multimedia Appendix 1; Figure S4D in Multimedia Appendix 2). This was the only outcome for which a significant time-by-group interaction was observed (*P*=.04), albeit in the unintended direction.

### Trial Retention

Over the course of the 12-month RCT, we saw steady rates of participant dropout, and only 67 participants (n=33, 49% in the intervention group and n=34, 51% in the control group) remained at the end of the 1-year study (retention rate=67/162, 41.4%). The greatest dropout rates were observed early in the trial between the week 2 and week 8 visits, followed by the periods later in the trial between the week 26 and week 39 visits and between the week 39 and week 52 visits.

### Dropout Analysis and Effect of COVID-19

**Figure 3:**
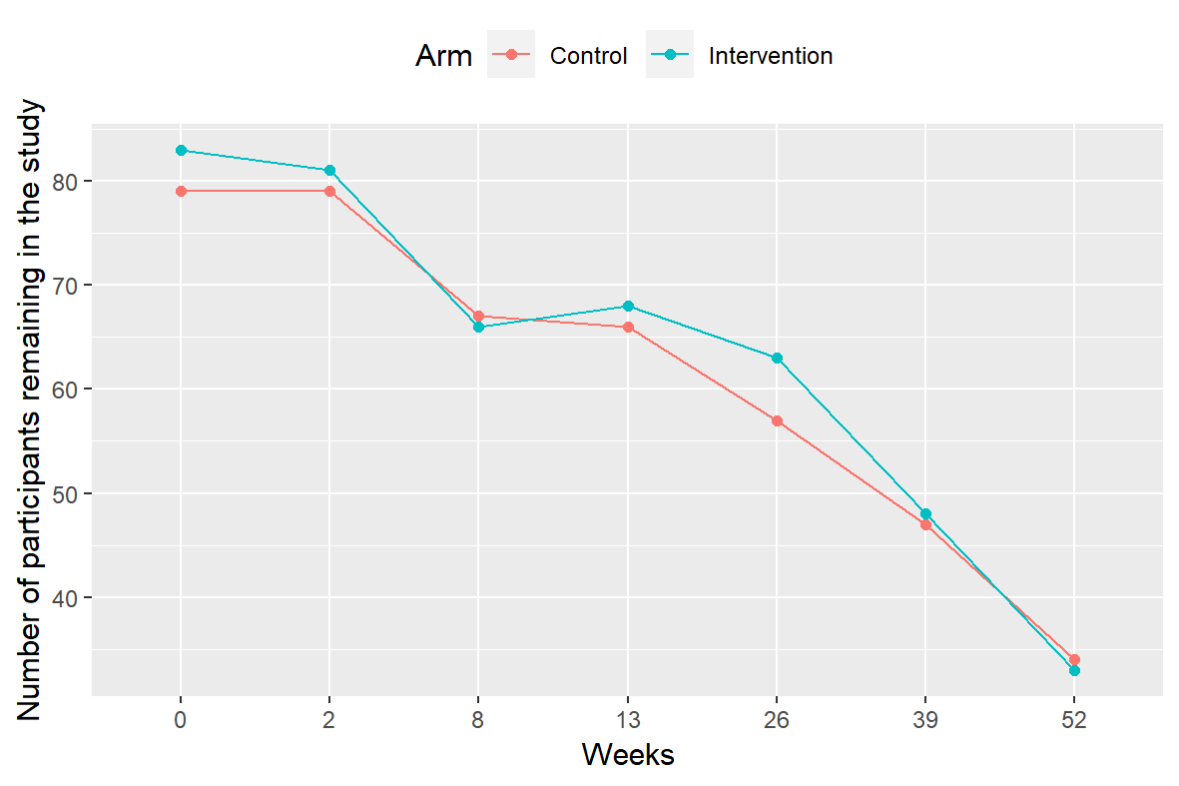
Dropout history over the course of the trial by study arm.

Steady dropout was observed in both treatment arms over the study period, amounting to 60% (50/83) and 57% (45/79) dropout in the MI-BP and control arms, respectively, at the end of study. These dropout patterns were very similar in both study arms (Figure 3). In the time-to-dropout analysis, marital status and COVID-19 group turned out to be statistically significant at the 5% level, with the post–COVID-19 phase showing a strong propensity (Table 4) for dropout (hazard ratio=2.12; SE 0.23; *P*=.001).

**Table 4:** Time-to-dropout results based on Cox regression model.

Variable		Hazard ratio (95% CI)	*P* value
COVID-19 group (reference: before COVID-19)		2.120 (1.353-3.322)	.001
Age		0.983 (0.959-1.008)	.18
**Marital status (reference: divorced, widowed, or separated)**			
	Married or cohabitating	1.998 (1.049-3.802)	.04
	Single or never been married	1.820 (1.021-3.247)	.04
Sex (reference: male)		0.912 (0.604-1.379)	.66

### AE Reporting

AEs for this trial were all determined to be cardiovascular in nature. A total of 15 AEs of SBP>180 were reported. All were determined to be unrelated to the study, and all were reported to both the WSU IRB and the study DSMB. In addition, during the course of this trial, 3 serious AEs (SAEs) were reported by research participants. All 3 SAEs were determined to be unexpected and unrelated to the MI-BP intervention, and all 3 patients recovered with treatment. SAEs reported in this trial included 2 instances of non–ST-elevation myocardial infarction and 1 instance of cerebral visual impairment. All SAEs were reported to both the WSU IRB and the study DSMB.

## Discussion

### Principal Findings

Results from this study suggest that, compared to our enhanced usual care control group, the MI-BP intervention did not have any significant effects on participants in this study, including the primary outcome measure of BP or the secondary outcome measures of physical activity, sodium intake, medication adherence, and BP control. Even though the trial was underpowered to detect differences due to stopping recruitment early because of the COVID-19 pandemic, trends to suggest that MI-BP had an effect on these outcomes compared to enhanced usual care were not evident. However, it is important to note that, despite a high overall dropout rate, participants in both groups experienced significant reductions in SBP, DBP, and sodium intake, as well as significant increases in physical activity and medication adherence, from baseline to 1 year. This speaks to the general benefit of proactive outreach and engagement focused on cardiometabolic risk reduction in urban-dwelling, low-socioeconomic-status Black populations.

Our findings are similar to those of the recent work by Pletcher et al [32], who found no benefit in terms of SBP reduction for BP self-monitoring using a connected smartphone app compared to standard BP self-monitoring over 6 months. As in our trial, Pletcher et al [32] found that in a sample of 2101 patients with uncontrolled BP, at 6 months, both the intervention and control arms experienced comparable and significant decreases in SBP (–10.8, SD 18 mm Hg vs –10.6, SD 18 mm Hg in the enhanced vs standard group, respectively) with no significant differences between the groups. While our findings and those of Pletcher et al [32] stand in contrast to those of other work suggesting benefit of mHealth apps in reducing BP [14], it should be noted that the evidence base for app-supported self-monitoring of BP is often plagued by short duration and follow-up, small sample sizes, and inconsistent comparison groups, which undermines the quality of research in this area. Moreover, it is important to remember that our control group was not assigned to usual care alone; rather, we used an active control condition where control group participants received a home BP monitor in addition to antihypertensive medication and standard educational materials. This may have led to greater reduction in BP than may have been experienced with standard usual care alone.

The high dropout rate (95/162, 58.6%) among enrolled and randomized participants warrants further mention. While this dropout rate is higher than those in other studies that involved similar digital interventions for monitoring and controlling BP or other study populations [33-35], high dropout rates in studies that deploy digital health interventions are quite common in the mHealth domain and range upward of 80% attrition, with approximately 49% attrition in observational studies and 40% attrition in RCTs. In his seminal piece, Eysenbach [36] described the law of attrition for eHealth interventions, which constitutes the phenomenon of participants dropping out of a research trial before completion or stopping their use of the trial intervention before the study is over. This phenomenon has been described time and time again in the digital health literature and has been specifically evident for mHealth interventions focused on physical activity [37,38], diet [39], and medication adherence (all targeted behaviors in the MI-BP intervention) [40]. Recent studies have demonstrated that a higher dropout rate is even more common for digital health intervention studies involving Black participants [41]. For instance, Jonassaint et al [42] suggested that it may be important to develop a digital intervention system that is culturally tailored to historically marginalized groups. However, our intervention was culturally tailored to the Black community, suggesting room for other explanations for the high dropout rate. We did include a wash-out period to screen out individuals who did not truly have uncontrolled hypertension and identify those who were not fully vested in trial participation, but neither of these is a culture-specific approach. Michaud et al [43] suggested that a high-incentive program may be effective in decreasing the attrition rate for digital health interventions for increasing physical activity in Black women. Although we incorporated a distributed incentive system, which rewarded participants for each visit completion, this was not sufficient to prevent the high dropout rates that echoed those in similar digital intervention studies. It should be mentioned that, particularly when working with historically excluded and underresourced communities, the notion of providing higher incentives to encourage participation is a hotly contested topic as some believe that it may be considered coercive; however, study incentives are meant to acknowledge participant burden, such as loss of time, and differentially incentivizing study participants based on level of advantage introduces its own host of ethical conundrums.

Our findings, coupled with the high dropout rates found in similar studies, suggest that these types of mHealth behavior change interventions may not be a complete solution that can promote behavior change and improve health outcomes in this population. Rather, mHealth may have the greatest potential as part of a suite of approaches available to health care professionals and patients. It is clear that mHealth solutions are here to stay, but the goal of future research should be to identify the use cases and implementation strategies and factors that contribute to optimization. Moreover, the expectation of high dropout rates for mHealth interventions, especially when working with challenging populations with considerable barriers caused by social determinants of health, should be assumed and addressed at the outset. This is important as underpowered samples compromise the quality of research studies and the evidence base; however, review panels for different funding mechanisms often look unfavorably on proposed research studies that anticipate very high dropout rates. This may cause researchers to intentionally underestimate attrition, which may compromise the research study as a whole.

### Limitations

As with all research, this study was not without limitations. Perhaps the largest limitation was the undeniable negative impact of the COVID-19 pandemic, which caused us to stop study recruitment early and likely had significant effects on trial participation for those who were already randomized to the study. Combined with a high dropout rate, early study termination led to an underpowered study, which may have caused us to be unable to find significant differences between the groups. That said, data trends across our participants suggest that both groups experienced significant improvement in both primary and secondary outcomes, and if there really was a benefit of the MI-BP intervention, it was likely much smaller than our initially projected effect size. Given the absence of an indication of differential improvements, future efforts would be better served simply focusing on scalable outreach and engagement programs that facilitate better care of Black patients with uncontrolled hypertension.

We should also note that, although we consented and enrolled 869 individuals in the ED and other community-based settings, 464 (53.4%) were considered lost to follow-up before randomization. Most of this loss to follow-up occurred before the baseline visit (Figure 1). Although we cannot say for certain why there was such tremendous loss to follow-up before randomization, our de facto wash-out period helped remove individuals who were not fully committed to participation in the trial. Given the large loss to follow-up before randomization, we most certainly had some degree of selection bias in our randomized sample. In addition to our loss to follow-up before randomization, as discussed, we experienced high dropout rates in our trial arms, which serves as a potential threat to the validity and generalizability of our findings. We also note that our quarterly follow-up assessments may have helped some participants stay engaged with the intervention and trial, masking even further attrition that may have been experienced without frequent contact. As suggested previously, very high rates of attrition are not uncommon in mHealth studies, and our trial was no exception. More research on how to best engage participants in these types of interventions and trials is desperately needed as the mechanisms that drive engagement are poorly understood. Moreover, our work was met with additional challenges as we are positioned in a community where social determinants of health play an enormous role in the daily lives of our participants. These additional challenges are sure to have contributed to our high attrition rates. While attrition may serve as a threat to the validity of our findings, it is important to remember that this is often the reality of working with populations of individuals who experience health inequity. Rather than shying away from work in this area due to methodological and statistical concerns, we must continue to conduct research with populations of individuals with great need to address inequities.

### Conclusions

Although we did not find increased improvement in outcomes for participants using MI-BP compared to enhanced usual care, we are encouraged to see that proactive outreach in a sample of Black individuals with uncontrolled hypertension recruited from EDs and other community-based settings had a significant effect in improving hypertension-related outcomes regardless of treatment group. Given that mHealth approaches for chronic disease self-management are becoming more commonplace, continued work is needed to understand how to better engage and retain users as well as how to better position these types of interventions within a suite of treatment options available to patients. Moreover, participant dropout in mHealth interventions remains high in many studies, including ours, and this phenomenon must be further explored before optimal mHealth use can be achieved.

## Appendix

Multimedia Appendix 1 Supplementary tables for primary and secondary outcomes.

Multimedia Appendix 2 Supplementary figures for secondary outcome trajectories over time.

Multimedia Appendix 3 CONSORT eHEALTH checklist (V 1.6.2).

## Abbreviations

AE: adverse event
ARMS-14: Adherence to Refills and Medication Scale
BP: blood pressure
BSS: Block Sodium Screener
CONSORT: Consolidated Standards of Reporting Trials
DBP: diastolic blood pressure
DSMB: Data and Safety Monitoring Board
ED: emergency department
HELM: Hypertension Evaluation of Lifestyle and Management
IPAQ-SF: International Physical Activity Questionnaire–Short Form
IRB: institutional review board
MASES: Medication Adherence Self-Efficacy Scale
MET: metabolic equivalent of task
mHealth: mobile health
PAM: Patient Activation Measure
PCP: primary care provider
RCT: randomized controlled trial
REALM-SF: Rapid Assessment of Adult Literacy in Medicine–Short Form
SAE: serious adverse event
SBP: systolic blood pressure
SF-12: Short Form–12
WSU: Wayne State University
