# Correction: The Effect of an mHealth Self-Monitoring Intervention (MI-BP) on Blood Pressure Among Black Individuals With Uncontrolled Hypertension: Randomized Controlled Trial

**DOI:** 10.2196/64632

**Published:** 2024-08-06

**Authors:** Lorraine R Buis, Junhan Kim, Ananda Sen, Dongru Chen, Katee Dawood, Reema Kadri, Rachelle Muladore, Melissa Plegue, Caroline R Richardson, Zora Djuric, Candace McNaughton, David Hutton, Lionel P Robert, Sun Young Park, Phillip Levy

**Affiliations:** 1 Department of Family Medicine University of Michigan Ann Arbor, MI United States; 2 School of Information University of Michigan Ann Arbor, MI United States; 3 Department of Biostatistics University of Michigan Ann Arbor, MI United States; 4 Department of Emergency Medicine and Integrative Biosciences Center Wayne State University Detroit, MI United States; 5 Department of Pediatrics University of Michigan Ann Arbor, MI United States; 6 Department of Family Medicine Brown University Providence, RI United States; 7 Department of Medicine Sunnybrook Research Institute University of Toronto Toronto, ON Canada; 8 School of Public Health University of Michigan Ann Arbor, MI United States

In “The Effect of an mHealth Self-Monitoring Intervention (MI-BP) on Blood Pressure Among Black Individuals With Uncontrolled Hypertension: Randomized Controlled Trial” (JMIR Mhealth Uhealth 2024;12:e57863) the authors noted an incorrect refernce [23]:

Marcus BH, Selby VC, Niaura RS, Rossi JS. Self-efficacy and the stages of exercise behavior change. Res Q Exerc Sport. Mar 1992;63(1):60-66.

The reference has been revised to:

Sallis J, Pinski R, Grossman R, Patterson T, Nader P. The development of self-efficacy scales for healthrelated diet and exercise behaviors. Health Educ Res. 1988;3(3):283-292.

The correction will appear in the online version of the paper on the JMIR Publications website on August 6, 2024, together with the publication of this correction notice. Because this was made after submission to PubMed, PubMed Central, and other full-text repositories, the corrected article has also been resubmitted to those repositories.
