# Supplementary material for: The Effect of an mHealth Self-Monitoring Intervention (MI-BP) on Blood Pressure Among Black Individuals With Uncontrolled Hypertension: Randomized Controlled Trial
Source: JMIR Mhealth Uhealth. 2024 Jun 28;12:e57863. doi: 10.2196/57863 (PMC11245662; doi:10.2196/57863)
Supplement: Multimedia Appendix 1 [file mhealth_v12i1e57863_app1.docx]

#### **Appendix 1: Supplementary Tables**

#### **Table S1: Regression coefficients and standard error for BP outcomes based on the LMM with linear time**

| **Variables** | **SBP** | | | **DBP** | | |
| --- | --- | --- | --- | --- | --- | --- |
|  | **Estimate** | **Standard Error** | **p-value** | **Estimate** | **Standard Error** | **p-value** |
| **Study Arm** (Ref: Control) | 0.8256 | 2.3043 | 0.7202 | 0.9543 | 1.3685 | 0.4858 |
| **Time** |  |  |  |  |  |  |
| Intervention | -0.2977 | 0.0506 | <.0001 | -0.1252 | 0.0269 | <.0001 |
| Control | -0.3351 | 0.0502 | <.0001 | -0.1727 | 0.0265 | <.0001 |
| **Time*Study Arm** | 0.0374 | 0.0712 | 0.5998 | 0.0475 | 0.0378 | 0.2093 |
| **Age** | -0.1920 | 0.1141 | 0.0930 | -0.4757 | 0.0693 | <.0001 |
| **Gender** (Ref: Male) | 0.2370 | 2.1182 | 0.9109 | -3.9321 | 1.2905 | 0.0024 |

**Table S2: Estimated pairwise mean differences and standard error across time for square-root transformed IPAQ-SF by Study Arm**

| **Comparison** | **Intervention** | | | **Control** | | |
| --- | --- | --- | --- | --- | --- | --- |
|  | **Mean Difference (time2-time1)** | **Standard Error** | **P-value** | **Mean Difference (time2-time1)** | **Standard Error** | **P-value** |
| 0-weeks vs. 13-weeks | 8.7717 | 4.6174 | 0.0586 | 5.7751 | 4.8677 | 0.2365 |
| 0-weeks vs. 26-weeks | 5.2379 | 4.8115 | 0.2773 | 7.5810 | 5.0749 | 0.1364 |
| 0-weeks vs. 39-weeks | 13.2226 | 5.1903 | 0.0114 | 9.7082 | 5.3283 | 0.0696 |
| 0-weeks vs. 52-weeks | 10.2516 | 6.3700 | 0.1088 | 10.5674 | 5.8727 | 0.0731 |
| 13-weeks vs. 26-weeks | -3.5338 | 4.9597 | 0.4768 | 1.8059 | 5.0007 | 0.7183 |
| 13-weeks vs. 39-weeks | 4.4509 | 5.3798 | 0.4088 | 3.9331 | 5.2014 | 0.4502 |
| 13-weeks vs. 52-weeks | 1.4799 | 6.5808 | 0.8222 | 4.7922 | 5.7680 | 0.4068 |
| 26-weeks vs. 39-weeks | 7.9847 | 5.5153 | 0.1489 | 2.1272 | 5.3369 | 0.6905 |
| 26-weeks vs. 52-weeks | 5.0137 | 6.6470 | 0.4514 | 2.9864 | 5.8780 | 0.6118 |
| 39-weeks vs. 52-weeks | -2.9710 | 6.9129 | 0.6677 | 0.8591 | 6.0221 | 0.8867 |

**Table S3: Estimated pairwise mean differences and standard error across time for square-root transformed BSS by Study Arm**

| **Comparison** | **Intervention** | | | **Control** | | |
| --- | --- | --- | --- | --- | --- | --- |
|  | **Mean Difference (time2-time1)** | **Standard Error** | **P-value** | **Mean Difference (time2-time1)** | **Standard Error** | **P-value** |
| 0-weeks vs. 13-weeks | -0.2288 | 0.1372 | 0.0963 | -0.4779 | 0.1403 | 0.0007 |
| 0-weeks vs. 26-weeks | -0.3390 | 0.1425 | 0.0178 | -0.1147 | 0.1489 | 0.4413 |
| 0-weeks vs. 39-weeks | -0.4303 | 0.1601 | 0.0075 | -0.5264 | 0.1628 | 0.0013 |
| 0-weeks vs. 52-weeks | -0.3572 | 0.1888 | 0.0593 | -0.5971 | 0.1846 | 0.0013 |
| 13-weeks vs. 26-weeks | -0.1103 | 0.1460 | 0.4507 | 0.3632 | 0.1521 | 0.0174 |
| 13-weeks vs. 39-weeks | -0.2015 | 0.1637 | 0.2191 | -0.0485 | 0.1650 | 0.7690 |
| 13-weeks vs. 52-weeks | -0.1284 | 0.1924 | 0.5050 | -0.1192 | 0.1865 | 0.5232 |
| 26-weeks vs. 39-weeks | -0.0913 | 0.1650 | 0.5804 | -0.4117 | 0.1690 | 0.0153 |
| 26-weeks vs. 52-weeks | -0.0182 | 0.1927 | 0.9250 | -0.4824 | 0.1890 | 0.0111 |
| 39-weeks vs. 52-weeks | 0.0731 | 0.2027 | 0.7185 | -0.0707 | 0.1966 | 0.7193 |

**Table S4: Estimated pairwise mean differences and standard error across time for log- transformed ARMS-14 by Study Arm**

| **Comparison** | **Intervention** | | | **Control** | | |
| --- | --- | --- | --- | --- | --- | --- |
|  | **Mean Difference (time2-time1)** | **Standard Error** | **P-value** | **Mean Difference (time2-time1)** | **Standard Error** | **P-value** |
| 0-weeks vs. 2-weeks | -0.0572 | 0.0283 | 0.0435 | -0.1309 | 0.0295 | <.0001 |
| 0-weeks vs. 8-weeks | -0.1133 | 0.0299 | 0.0002 | -0.1467 | 0.0311 | <.0001 |
| 0-weeks vs. 13-weeks | -0.1170 | 0.0301 | 0.0001 | -0.1871 | 0.0322 | <.0001 |
| 0-weeks vs. 26-weeks | -0.1110 | 0.0318 | 0.0005 | -0.1331 | 0.0334 | <.0001 |
| 0-weeks vs. 39-weeks | -0.1325 | 0.0342 | 0.0001 | -0.1719 | 0.0361 | <.0001 |
| 0-weeks vs. 52-weeks | -0.1979 | 0.0415 | <.0001 | -0.1520 | 0.0405 | 0.0002 |
| 2-weeks vs. 8-weeks | -0.0561 | 0.0301 | 0.0625 | -0.0158 | 0.0301 | 0.6001 |
| 2-weeks vs. 13-weeks | -0.0598 | 0.0303 | 0.0483 | -0.0562 | 0.0313 | 0.0725 |
| 2-weeks vs. 26-weeks | -0.0538 | 0.0319 | 0.0922 | -0.0023 | 0.0327 | 0.9444 |
| 2-weeks vs. 39-weeks | -0.0754 | 0.0343 | 0.0282 | -0.0410 | 0.0356 | 0.2493 |
| 2-weeks vs. 52-weeks | -0.1407 | 0.0416 | 0.0008 | -0.0211 | 0.0400 | 0.5978 |
| 8-weeks vs. 13-weeks | -0.0037 | 0.0314 | 0.9055 | -0.0404 | 0.0322 | 0.2097 |
| 8-weeks vs. 26-weeks | 0.0023 | 0.0332 | 0.9455 | 0.0135 | 0.0335 | 0.6869 |
| 8-weeks vs. 39-weeks | -0.0192 | 0.0352 | 0.5852 | -0.0252 | 0.0362 | 0.4867 |
| 8-weeks vs. 52-weeks | -0.0846 | 0.0425 | 0.0468 | -0.0053 | 0.0406 | 0.8961 |
| 13-weeks vs. 26-weeks | 0.0060 | 0.0330 | 0.8558 | 0.0540 | 0.0344 | 0.1171 |
| 13-weeks vs. 39-weeks | -0.0155 | 0.0353 | 0.6600 | 0.0152 | 0.0371 | 0.6812 |
| 13-weeks vs. 52-weeks | -0.0809 | 0.0426 | 0.0583 | 0.0351 | 0.0415 | 0.3973 |
| 26-weeks vs. 39-weeks | -0.0215 | 0.0364 | 0.5549 | -0.0387 | 0.0378 | 0.3063 |
| 26-weeks vs. 52-weeks | -0.0869 | 0.0435 | 0.0463 | -0.0188 | 0.0419 | 0.6538 |
| 39-weeks vs. 52-weeks | -0.0654 | 0.0449 | 0.1457 | 0.0199 | 0.0438 | 0.6502 |

**Table S5: Estimated pairwise mean differences and standard error across time for Self-Efficacy for Exercise Behaviors (SEEB) *Sticking to It* subscale by Study Arm**

| **Comparison** | **Intervention** | | | **Control** | | |
| --- | --- | --- | --- | --- | --- | --- |
|  | **Mean Difference (time2-time1)** | **Standard Error** | **P-value** | **Mean Difference (time2-time1)** | **Standard Error** | **P-value** |
| 0-weeks vs. 13-weeks | -0.4644 | 0.1437 | 0.0013 | -0.2453 | 0.1457 | 0.0930 |
| 0-weeks vs. 26-weeks | -0.3284 | 0.1503 | 0.0295 | -0.2326 | 0.1535 | 0.1307 |
| 0-weeks vs. 39-weeks | -0.1927 | 0.1656 | 0.2453 | -0.0772 | 0.1655 | 0.6412 |
| 0-weeks vs. 52-weeks | -0.1078 | 0.1996 | 0.5896 | -0.0149 | 0.1834 | 0.9351 |
| 13-weeks vs. 26-weeks | 0.1360 | 0.1564 | 0.3852 | 0.0127 | 0.1581 | 0.9359 |
| 13-weeks vs. 39-weeks | 0.2716 | 0.1712 | 0.1135 | 0.1681 | 0.1696 | 0.3223 |
| 13-weeks vs. 52-weeks | 0.3566 | 0.2044 | 0.0819 | 0.2303 | 0.1871 | 0.2190 |
| 26-weeks vs. 39-weeks | 0.1356 | 0.1746 | 0.4376 | 0.1553 | 0.1739 | 0.3722 |
| 26-weeks vs. 52-weeks | 0.2206 | 0.2067 | 0.2866 | 0.2176 | 0.1902 | 0.2534 |
| 39-weeks vs. 52-weeks | 0.0850 | 0.2161 | 0.6944 | 0.0623 | 0.1971 | 0.7522 |

**Table S6: Estimated pairwise mean differences and standard error across time for Self-Efficacy for Exercise Behaviors (SEEB) *Making Time for Exercise* subscale by Study Arm**

| **Comparison** | **Intervention** | | | **Control** | | |
| --- | --- | --- | --- | --- | --- | --- |
|  | **Mean Difference (time2-time1)** | **Standard Error** | **P-value** | **Mean Difference (time2-time1)** | **Standard Error** | **P-value** |
| 0-weeks vs. 13-weeks | -0.4373 | 0.1572 | 0.0057 | -0.3875 | 0.1609 | 0.0165 |
| 0-weeks vs. 26-weeks | -0.4587 | 0.1652 | 0.0058 | -0.2899 | 0.1696 | 0.0883 |
| 0-weeks vs. 39-weeks | -0.2071 | 0.1838 | 0.2606 | -0.2323 | 0.1815 | 0.2013 |
| 0-weeks vs. 52-weeks | -0.0857 | 0.2177 | 0.6942 | -0.1120 | 0.2031 | 0.5816 |
| 13-weeks vs. 26-weeks | -0.0214 | 0.1717 | 0.9009 | 0.0977 | 0.1745 | 0.5761 |
| 13-weeks vs. 39-weeks | 0.2302 | 0.1897 | 0.2256 | 0.1552 | 0.1855 | 0.4034 |
| 13-weeks vs. 52-weeks | 0.3517 | 0.2225 | 0.1149 | 0.2755 | 0.2066 | 0.1831 |
| 26-weeks vs. 39-weeks | 0.2516 | 0.1944 | 0.1963 | 0.0575 | 0.1906 | 0.7629 |
| 26-weeks vs. 52-weeks | 0.3731 | 0.2263 | 0.1000 | 0.1779 | 0.2104 | 0.3984 |
| 39-weeks vs. 52-weeks | 0.1214 | 0.2379 | 0.6100 | 0.1203 | 0.2175 | 0.5803 |

**Table S7: Estimated pairwise mean differences and standard error across time for MASES by Study Arm**

| **Comparison** | **Intervention** | | | **Control** | | |
| --- | --- | --- | --- | --- | --- | --- |
|  | **Mean Difference (time2-time1)** | **Standard Error** | **P-value** | **Mean Difference (time2-time1)** | **Standard Error** | **P-value** |
| 0-weeks vs. 13-weeks | -0.0984 | 0.1182 | 0.4054 | 0.0792 | 0.1201 | 0.5101 |
| 0-weeks vs. 26-weeks | -0.1854 | 0.1234 | 0.1338 | 0.1063 | 0.1272 | 0.4037 |
| 0-weeks vs. 39-weeks | 0.2772 | 0.1354 | 0.0413 | 0.3583 | 0.1350 | 0.0083 |
| 0-weeks vs. 52-weeks | 0.3843 | 0.1616 | 0.0179 | 0.3713 | 0.1523 | 0.0152 |
| 13-weeks vs. 26-weeks | -0.0869 | 0.1272 | 0.4949 | 0.0272 | 0.1305 | 0.8353 |
| 13-weeks vs. 39-weeks | 0.3757 | 0.1391 | 0.0072 | 0.2791 | 0.1382 | 0.0441 |
| 13-weeks vs. 52-weeks | 0.4827 | 0.1648 | 0.0036 | 0.2921 | 0.1551 | 0.0604 |
| 26-weeks vs. 39-weeks | 0.4626 | 0.1416 | 0.0012 | 0.2520 | 0.1424 | 0.0775 |
| 26-weeks vs. 52-weeks | 0.5697 | 0.1668 | 0.0007 | 0.2650 | 0.1583 | 0.0949 |
| 39-weeks vs. 52-weeks | 0.1071 | 0.1744 | 0.5396 | 0.0130 | 0.1631 | 0.9367 |

**Table S8: Estimated pairwise mean differences and standard error across time for Self-Efficacy for Eating Behaviors by Study Arm**

| **Comparison** | **Intervention** | | | **Control** | | |
| --- | --- | --- | --- | --- | --- | --- |
|  | **Mean Difference (time2-time1)** | **Standard Error** | **P-value** | **Mean Difference (time2-time1)** | **Standard Error** | **P-value** |
| 0-weeks vs. 13-weeks | -0.8398 | 1.0662 | 0.4314 | 1.6774 | 1.0875 | 0.1238 |
| 0-weeks vs. 26-weeks | -0.5924 | 1.1044 | 0.5920 | 0.2719 | 1.1423 | 0.8120 |
| 0-weeks vs. 39-weeks | -1.1451 | 1.2339 | 0.3540 | 1.5844 | 1.2340 | 0.1999 |
| 0-weeks vs. 52-weeks | -3.9065 | 1.4675 | 0.0081 | 2.0539 | 1.3991 | 0.1429 |
| 13-weeks vs. 26-weeks | 0.2474 | 1.1444 | 0.8290 | -1.4055 | 1.1757 | 0.2327 |
| 13-weeks vs. 39-weeks | -0.3053 | 1.2709 | 0.8103 | -0.0930 | 1.2597 | 0.9412 |
| 13-weeks vs. 52-weeks | -3.0667 | 1.5042 | 0.0421 | 0.3766 | 1.4229 | 0.7914 |
| 26-weeks vs. 39-weeks | -0.5527 | 1.2824 | 0.6667 | 1.3125 | 1.2865 | 0.3083 |
| 26-weeks vs. 52-weeks | -3.3141 | 1.5081 | 0.0286 | 1.7820 | 1.4398 | 0.2166 |
| 39-weeks vs. 52-weeks | -2.7614 | 1.5830 | 0.0819 | 0.4696 | 1.4868 | 0.7523 |
