# Supplementary material for: The Effect of an mHealth Self-Monitoring Intervention (MI-BP) on Blood Pressure Among Black Individuals With Uncontrolled Hypertension: Randomized Controlled Trial
Source: JMIR Mhealth Uhealth. 2024 Jun 28;12:e57863. doi: 10.2196/57863 (PMC11245662; doi:10.2196/57863)
Supplement: Multimedia Appendix 2 [file mhealth_v12i1e57863_app2.docx]

**Appendix 2: Supplementary Figures**

**Figure S1: Unadjusted mean trajectories (with 95% confidence interval) for physical activity (iPAQ-SF) by study arm**


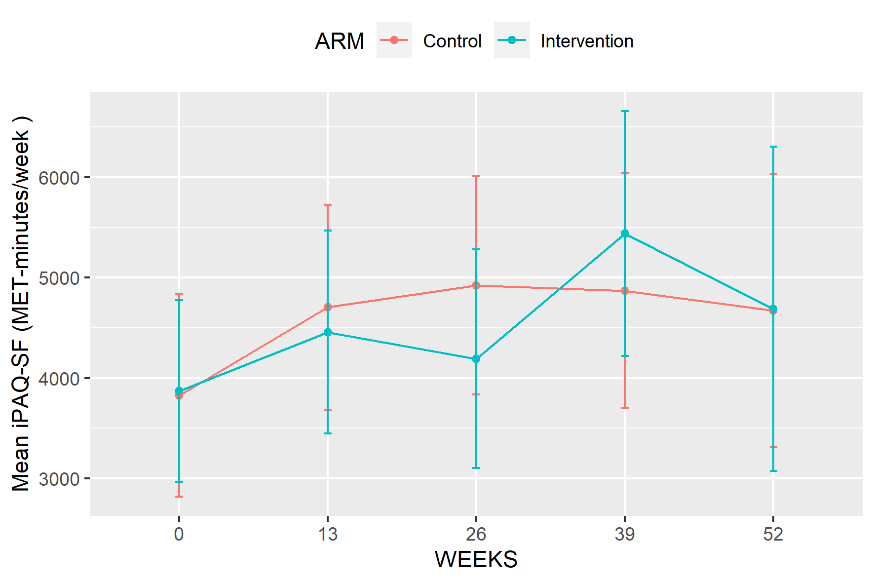


**Figure S2: Unadjusted mean trajectories (with 95% confidence interval) for sodium (BSS) by study arm**


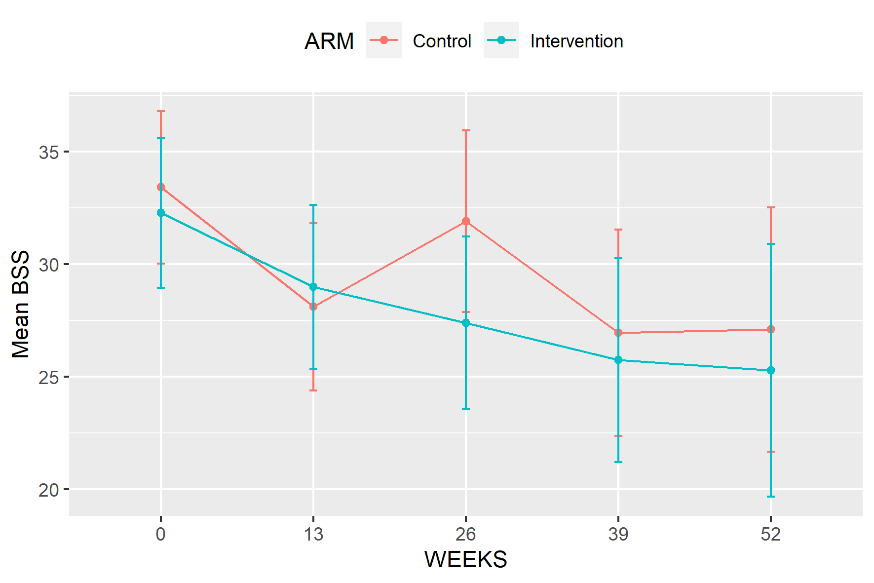


**Figure S3: Unadjusted mean trajectories (with 95% confidence interval) for medication adherence (ARMS-14) by study arm**


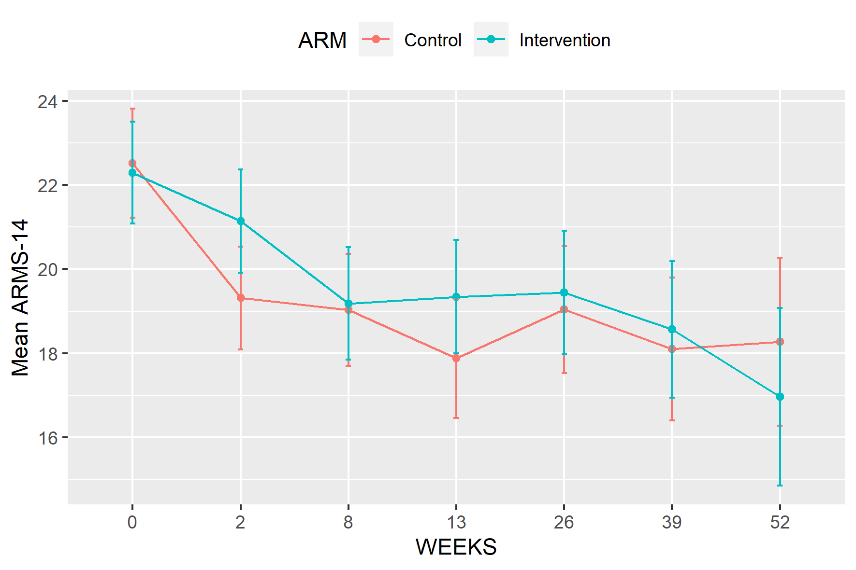


**Figure S4: Unadjusted mean trajectories (with 95% confidence interval) for self-efficacy outcomes by study arm**

**
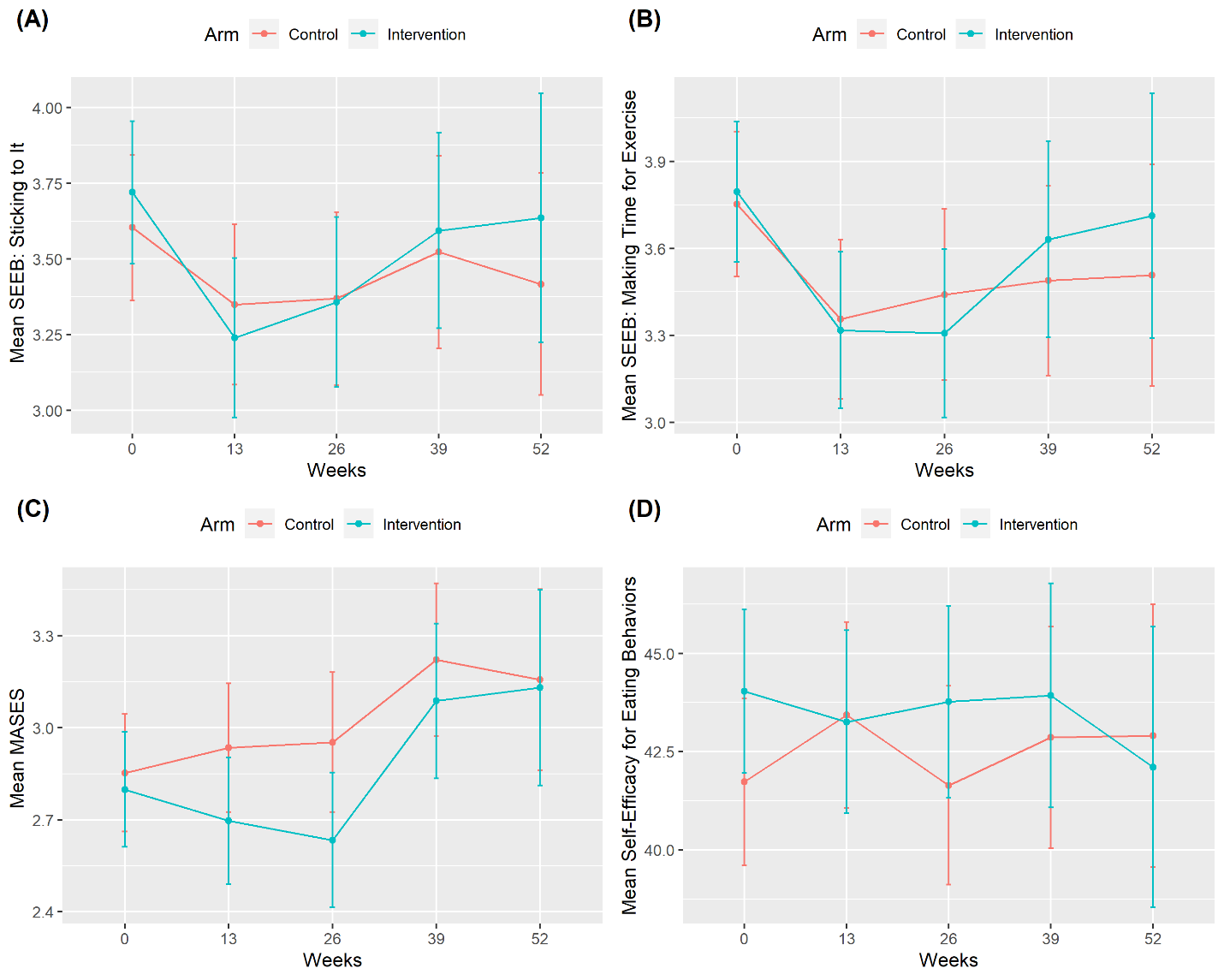
**
